# A Complex Differential for Gastric Outlet Obstruction: Bezoar or Bouveret?

**DOI:** 10.7759/cureus.92412

**Published:** 2025-09-15

**Authors:** Leo Sakai, Jadon Styadi, Kristelle Imperio-Lagabon, Daniel Chao

**Affiliations:** 1 Internal Medicine, Loma Linda University Medical Center, Loma Linda, USA; 2 Medicine, Loma Linda University School of Medicine, Loma Linda, USA; 3 Gastroenterology and Hepatology, Loma Linda University Medical Center, Loma Linda, USA; 4 Gastroenterology and Hepatology, Veterans Affairs Medical Center, Loma Linda, USA

**Keywords:** abdominal pain with nausea and vomiting, a case report, bouveret's syndrome, dysmotility, gastric outlet obstruction (goo), gastroenterology and endoscopy, phyto-trichobezoar

## Abstract

Bezoars are a rare but important cause of gastric outlet obstruction (GOO). Recognizing this diagnosis promptly is important to prevent complications, such as ulceration, bleeding, and perforation. Typical presenting symptoms include postprandial nausea, vomiting, and epigastric abdominal pain. However, these findings can be non-specific and imitate other rare causes of GOO. Here, we describe a case of a 77-year-old male who presented with GOO due to a phytobezoar mimicking Bouveret’s syndrome. This case highlights the diagnostic and therapeutic challenges of GOO.

## Introduction

Gastric outlet obstruction (GOO) is a clinical syndrome caused by an inability of gastric contents to pass through the pylorus, often presenting with postprandial nausea, vomiting, and epigastric abdominal pain [1]. Presenting symptoms can often be nonspecific; therefore, it is critical to implement various diagnostic modalities, including imaging, endoscopy, and blood tests, to reach an accurate and prompt diagnosis. Obtaining a thorough medication history is also crucial, as various pharmacological agents can increase the risk of GOO [2]. The etiologies of GOO are diverse and include peptic ulcer disease with inflammatory edema or stricture formation; primary gastric neoplasm such as adenocarcinoma, lymphoma, and neuroendocrine tumor; invasive malignancy from adjacent structures such as the gallbladder or pancreas; pancreatic fluid collections; and less commonly gastric involvement of Crohn’s disease, tuberculosis, an impacted gallstone (Bouveret syndrome), and bezoars [3]. We report an interesting case of GOO secondary to a phytobezoar presenting with findings concerning Bouveret syndrome.

## Case presentation

A 77-year-old male with chronic low back pain requiring chronic opioid use, longstanding diabetes, cholelithiasis, a history of exploratory laparotomy and splenectomy following a motor vehicle accident 36 years ago, and diffuse large B-cell lymphoma (DLBCL) of the stomach treated with chemoradiation in clinical remission for the past seven years presented with intermittent epigastric pain, nausea, vomiting, early satiety, and 60 pounds of unintentional weight loss over the past seven months. His symptoms became progressively worse over a few days’ time, prompting him to visit the Emergency Department (ED).

On admission, vital signs were normal. Review of systems was negative for hematemesis, hematochezia, and melena. His medications were significant for naproxen and hydrocodone. Physical exam was notable for epigastric tenderness. The abdomen was nondistended, and bowel sounds were present. Labs on admission were remarkable for mild hyponatremia and hypochloremia, with liver function test, amylase, and lipase within normal range (Table 1). 

**Table 1:** Laboratory results on admission µL: microliter; g: gram; dL: deciliter; mMol: millimole; mg: milligram; L: liter; IU: international units; U: units

Analyte	Results	Reference Range
Complete Blood Count		
White blood cell count	10.86 K/uL	4.0-10.0 K/uL
Hemoglobin	13.7 g/dL	13.5-17.5 g/dL
Hematocrit	40.00%	40.0-53.0%
Platelet count	296 K/uL	150-450 K/uL
Complete Metabolic Panel		
Sodium	130 mMol/L	136-144 mMol/L
Potassium	4.2 mMol/L	3.6-5.1 mMol/L
Chloride	94 mMol/L	101-111 mMol/L
Bicarbonate	25.0 mMol/L	22-32 mMol/L
Blood urea nitrogen	15 mg/dL	8-20 mg/dL
Creatinine	0.97 mg/dL	0.64-1.27 mg/dL
Glucose	126 mg/dL	74-118 mg/dL
Liver Function Test		
Aspartate aminotransferase	16 IU/L	15-41 IU/L
Alanine aminotransferase	15 IU/L	17-63 IU/L
Alkaline phosphatase	80 IU/L	32-91 IU/L
Total bilirubin	1.0 mg/dL	0.2-1.2 mg/dL
Albumin	3.7 gm/dL	3.5-4.8 gm/dL
Miscellaneous		
Amylase	28 IU/L	28-100 IU/L
Lipase	22 U/L	22-51 U/L

**Figure 1:**
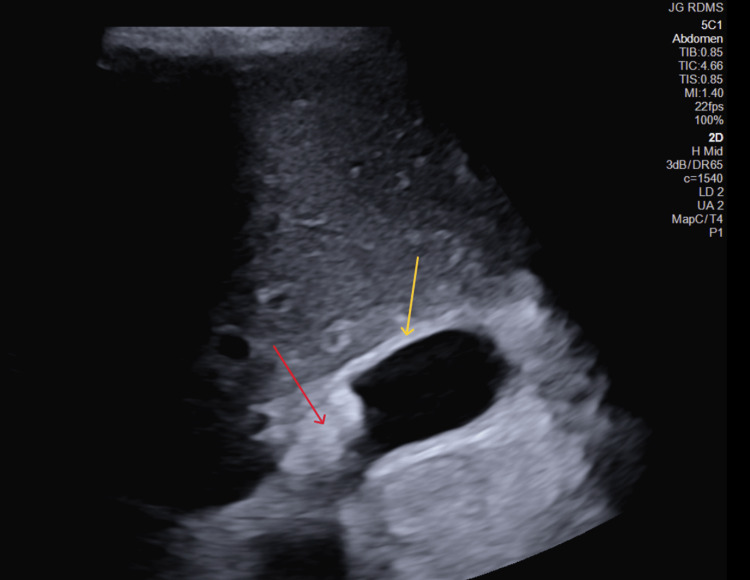
Abdominal ultrasound Red arrow: mobile cholelithiasis; yellow arrow: gallbladder wall thickening measuring >3 mm

Abdominal ultrasound (US) showed multiple gallstones with gallbladder wall thickening (Figure 1). Sonographic Murphy’s sign was positive.

**Figure 2:**
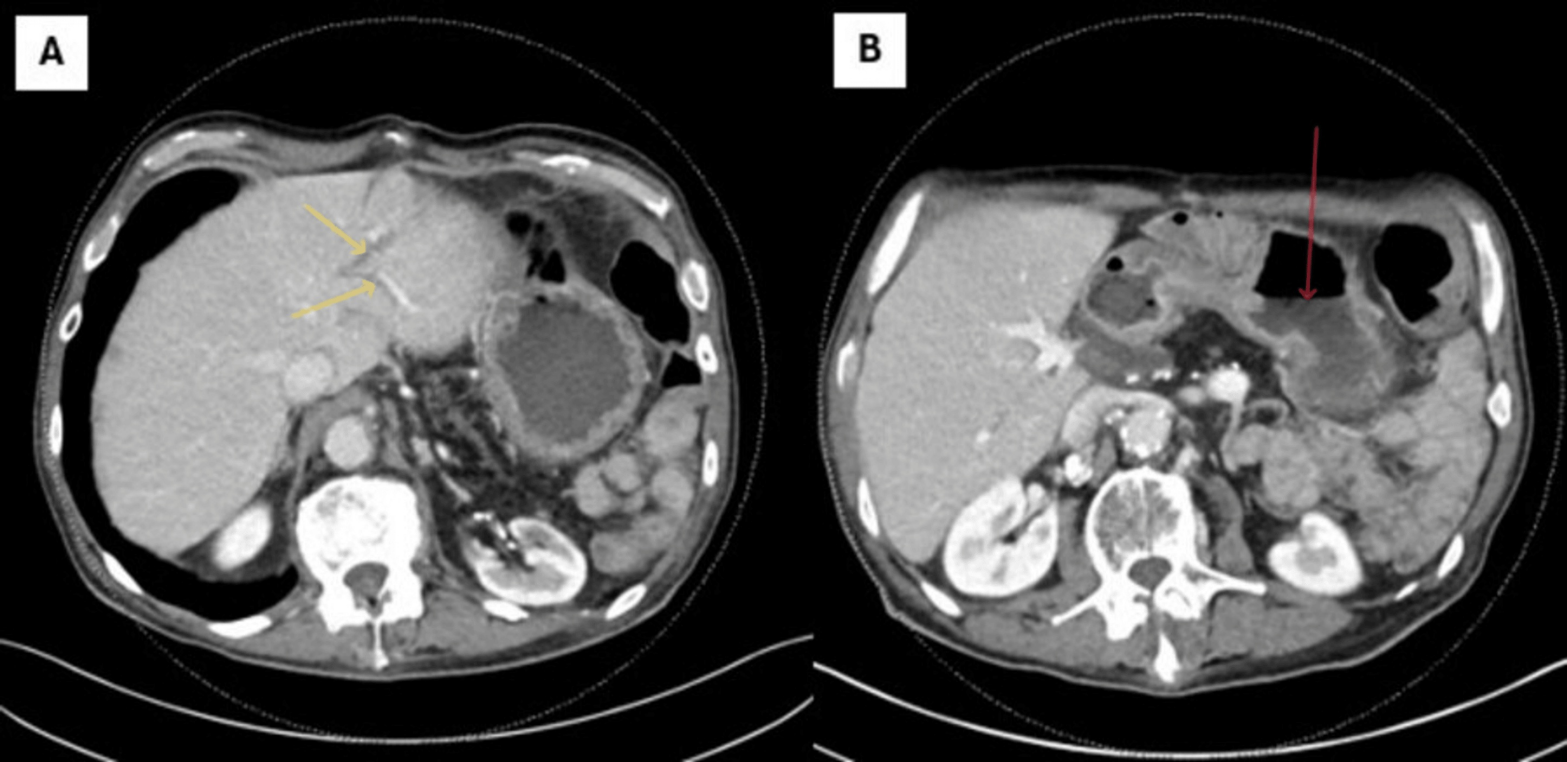
A) New mildly dilated intrahepatic bile ducts on axial CT of the abdomen. B) Retained gastric content and air fluid level Yellow arrow: dilated intrahepatic ducts; Red arrow: retained gastric contents

Computed tomography (CT) of the abdomen/pelvis revealed intrahepatic and common bile duct dilatation (Figure 2A), retained gastric contents with an air fluid level, and duodenal wall thickening (Figure 2B), concerning for an obstructive process.

**Figure 3:**
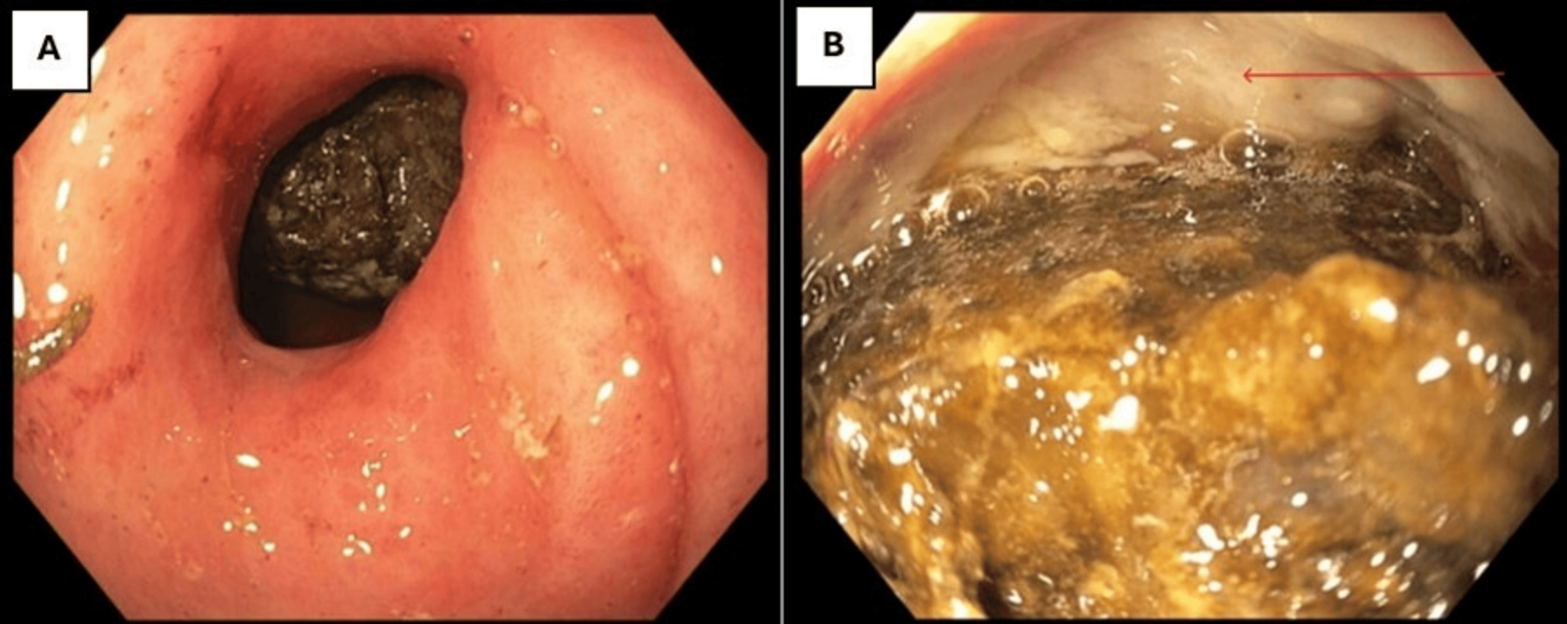
A) Narrowed and inflamed pylorus that resisted the passage of the endoscope with a foreign body visible in the duodenal bulb. B) Large, obstructing foreign body in the duodenal bulb and ulcer at the 12 o'clock position Red arrow: clean-based ulcer

The patient underwent an esophagogastroduodenoscopy (EGD), which revealed a stiff, narrowed pylorus that resisted passage. In the duodenal bulb, a large, 20 mm, yellow-brown foreign body obstructed the entrance to the sweep. The object was hard and round with an irregular border. Distal to the foreign body, a clean-based ulcer involving 50% of the circumference was visualized (Figures 3A-3B). 

**Figure 4:**
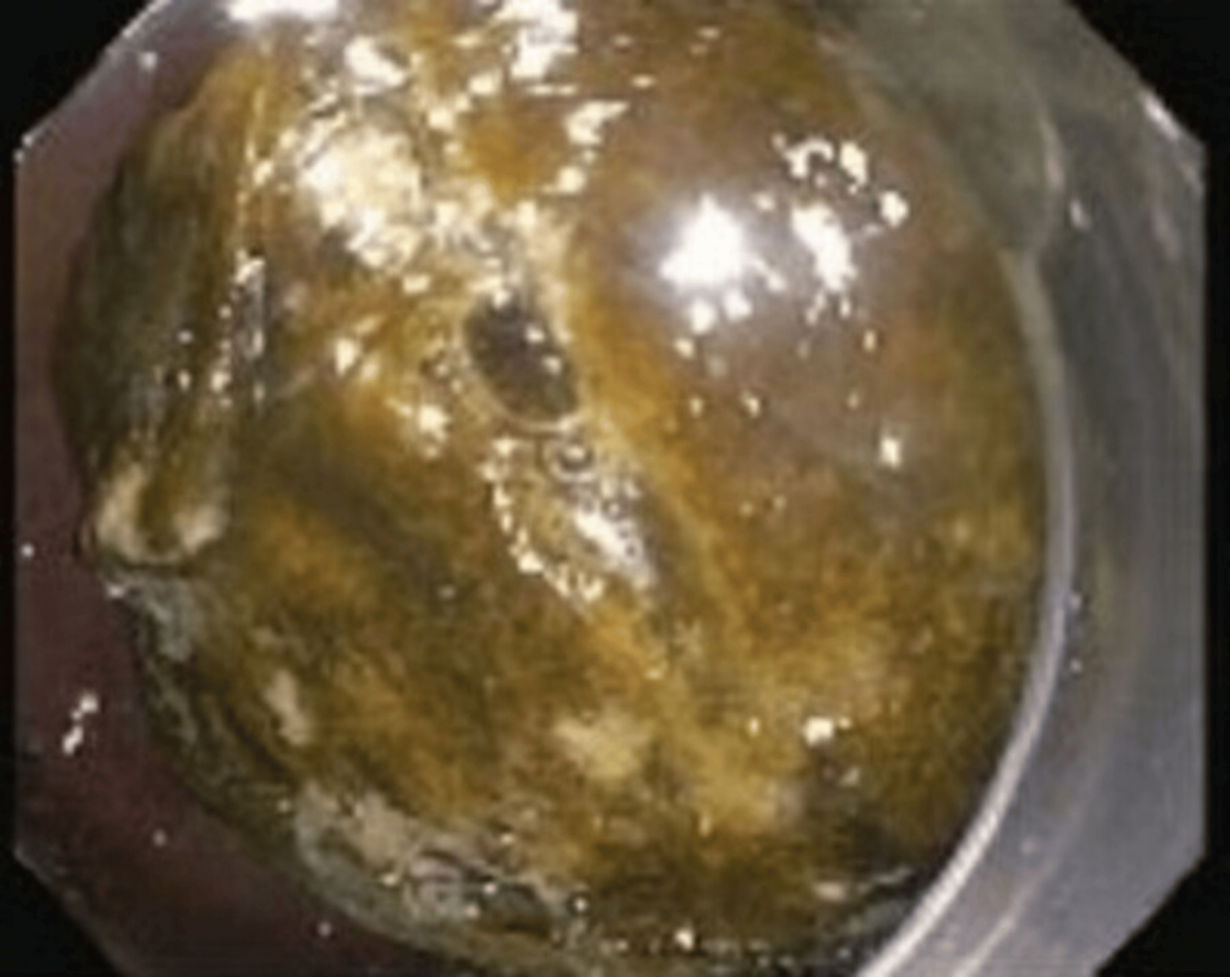
Large foreign object in the duodenal bulb was re-demonstrated on repeat endoscopy by interventional gastroenterology

Multiple attempts to extract the foreign body using a retrieval net, rat tooth forceps, and a snare were unsuccessful. Given the dilated bile ducts and gallstones seen on imaging, there was concern that the foreign body was a migrated gallstone, possibly with food packed on top. EGD was repeated by interventional gastroenterology, and removal of the foreign body was attempted using an electrohydraulic lithotripter (Figure 4) and rat tooth forceps, but the material eventually became too hard to continue. The patient underwent pyloromyotomy with successful extraction of the green-brown material measuring 3.8 x 2.1 x 1.8 cm. Pathological analysis revealed plant-like material, confirming the diagnosis of phytobezoar.

## Discussion

A bezoar is a collection of undigestible material in the GI tract that can cause GOO if impacted in the antrum or duodenum. Bezoars are classified by composition: phytobezoar (plant material), trichobezoar (hair), pharmacobezoar (medications), lactobezoar (milk protein), and lithobezoar (ingested stones) [3]. The incidence and prevalence of bezoars are unknown, with no population studies found in the literature at this time. It is an infrequent finding, with one retrospective study from 1978 reporting an incidence of 0.3% [4].

Although bezoars can form in individuals with a normally functioning GI tract, those with abnormal GI motility and decreased gastric acid secretion are at increased risk. Therefore, risk factors include prior gastric surgery, gastroparesis, use of anticholinergic or opioid medications, use of bulky medication (especially in combination with anti-secretory medications), hypothyroidism, neurodegenerative conditions, and dehydration [5].

The patient in this case had several risk factors for developing a bezoar, including longstanding diabetes and chronic opioid use [6]. Although our patient had no history of gastric surgery, he had a history of DLBCL involving the stomach treated with radiation, both of which can alter gastric motility [7,8]. His prior laparotomy could have caused delayed gastric emptying due to vagal injury.

The diagnosis of a bezoar is usually made via a CT scan or EGD. The former is often performed in the Emergency Room setting when patients present with symptoms of GOO. Findings on CT include gastric distention, retained gastric contents, and an air fluid level [9]. EGD allows for direct visualization of the bezoar and assessment of mechanical risk factors for obstruction.

What makes this case unique is that the clinical presentation mimicked Bouveret syndrome. Bouveret syndrome occurs when a gallstone migrates to the duodenum through a bilioduodenal fistula [10] and becomes impacted, leading to proximal small bowel obstruction and similar symptoms of GOO. The clinical findings for Bouveret syndrome are non-specific, and physical exam findings can be subtle, making this a challenging diagnosis [10]. This clinical entity should be considered in patients with a history of cholelithiasis presenting with symptoms of bowel obstruction.

Abdominal X-ray may show the classic Rigler’s triad for Bouveret syndrome: a dilated stomach, pneumobilia, and ectopic gallstones. This constellation of findings has reasonable specificity, but sensitivity of only 15%-20%. Abdominal CT had the highest rate of demonstrating Rigler’s triad but is not adept at identifying stones. Magnetic resonance cholangiopancreatography (MRCP) is able to provide a highly sensitive and specific evaluation of the bile ducts, including the cystic duct, and can identify fistulas. EGD, like for bezoars, allows for direct visualization of the obstruction and impacted stone [11].

The clinical features in this case that raised concern for Bouveret syndrome included the presence of cholelithiasis, sonographic Murphy’s sign, gallbladder wall thickening, and biliary ductal dilatations. It is possible that the patient's biliary tract dilation was related to chronic opioid use, but the progressive involvement of the intrahepatic ducts was concerning for a more active process at the time of evaluation. The impacted foreign body found on EGD had some resemblance to a stone, and the overlying vegetable matter could have gathered later. No fistula was seen, which is an important pertinent negative, but the literature has reported multiple instances of Bouveret syndrome where endoscopy failed to reveal the bilioduodenal fistula. During surgery, the serosal surface of the gallbladder was observed to be intact, supporting the diagnosis of bezoars over Bouveret syndrome.

The management of bezoars should be tailored to the type and composition of the mass. For those who can tolerate oral intake and have a bezoar made of dissolvable materials, chemical dissolution may be an option. There is no standard approach, but combinations of cellulase, cysteine, and metoclopramide have been reported, as has the use of Coca-Cola due to its acidic properties [12,13]. Endoscopic intervention is appropriate for patients with obstruction severe enough that oral intake is compromised, those with bleeding from mucosal injury and ulceration, and when chemical dissolution fails [14,15]. Surgical extraction is indicated in patients who haven’t responded to these measures or if there are signs of bowel perforation. Despite treatment, up to 20% of patients with bezoars experience recurrent episodes. Depending on the type of bezoar, increasing water intake, dietary modification, avoidance of certain medications, and correction of underlying motility disorders may help reduce the risk of recurrence.

In this patient, chemical dissolution was not a reasonable approach due to the severity of the GOO, which made prompt endoscopic evaluation indicated. As discussed, the composition of the bezoar was not clear until post-surgical analysis confirmed that there was only plant-like material. Endoscopic extraction of the bezoar was attempted twice but was ultimately unsuccessful, necessitating surgical extraction.

## Conclusions

Bezoars are a rare but important cause of GOO. Recognizing this diagnosis promptly is vital to prevent complications such as bleeding and perforation. It can mimic other causes of GOO, such as Bouveret syndrome, as demonstrated in our patient. The patient had several risk factors that predisposed him to bezoar formation, so he may need to be mindful of his fiber intake. Follow-up should include endoscopic surveillance to ensure that his gastric lymphoma is still in remission.
